# Returning a lost process by reintroducing a locally extinct digging marsupial

**DOI:** 10.7717/peerj.6622

**Published:** 2019-05-27

**Authors:** Nicola T. Munro, Sue McIntyre, Ben Macdonald, Saul A. Cunningham, Iain J. Gordon, Ross B. Cunningham, Adrian D. Manning

**Affiliations:** 1 Fenner School of Environment and Society, Australian National University, Canberra, ACT, Australia; 2 Land and Water, Commonwealth Scientific and Industrial Research Organisation, Canberra, ACT, Australia; 3 Division of Tropical Environments and Societies, James Cook University of North Queensland, Townsville, QLD, Australia; 4 James Hutton Institute, Craigiebuckler, Aberdeen, UK

**Keywords:** Soil processes, Restoration, Reintroduction, Bioturbation, Ecosystem engineering

## Abstract

The eastern bettong (*Bettongia gaimardi*), a medium-sized digging marsupial, was reintroduced to a predator-free reserve after 100 years of absence from the Australian mainland. The bettong may have the potential to restore temperate woodlands degraded by a history of livestock grazing, by creating numerous small disturbances by digging. We investigated the digging capacity of the bettong and compared this to extant fauna, to answer the first key question of whether this species could be considered an ecosystem engineer, and ultimately if it has the capacity to restore lost ecological processes. We found that eastern bettongs were frequent diggers and, at a density of 0.3–0.4 animals ha^−1^, accounted for over half the total foraging pits observed (55%), with echidnas (*Tachyglossus aculeatus*), birds and feral rabbits (*Oryctolagus cuniculus*) accounting for the rest. We estimated that the population of bettongs present dug 985 kg of soil per ha per year in our study area. Bettongs dug more where available phosphorus was higher, where there was greater basal area of *Acacia* spp. and where kangaroo grazing was less. There was no effect on digging of eucalypt stem density or volume of logs on the ground. While bettong digging activity was more frequent under trees, digging also occurred in open grassland, and bettongs were the only species observed to dig in scalds (areas where topsoil has eroded to the B Horizon). These results highlight the potential for bettongs to enhance soil processes in a way not demonstrated by the existing fauna (native birds and echidna), and introduced rabbit.

## Introduction

The restoration of ecosystems must include the restoration of functions and processes (Bennett et al., 2009; Hobbs & Harris, 2001; Prober et al., 2005). The reinstatement of lost species that perform key functions, for example, ‘ecosystem engineers’ (sensu Jones et al., 2010; Jones, Lawton & Shachak, 1994), has been advocated as an important tool for ecological restoration (Eldridge & James, 2009; Fleming et al., 2014; Hobbs & Cramer, 2008; Martin, 2003). In the specific function of soil engineering through digging, animals can alter soil processes, for example, by breaking up cryptogamic crusts and increasing water infiltration (Eldridge & Mensinga, 2007; Garkaklis, Bradley & Wooller, 1998), increasing nutrient cycling (Eldridge & James, 2009; James & Eldridge, 2007; Travers et al., 2012), reducing bulk density of soils (Fleming et al., 2014; Travers et al., 2012), and creating pits that act as traps for seed, leaf litter and sediment, which then become foci for seed germination (Eldridge & James, 2009; Gutterman, Golan & Garsani, 1990; James, Eldridge & Hill, 2009; Valentine et al., 2017). Digging can also alter plant dynamics by reducing plant competition through the creation of gaps allowing plant recruitment (Sandom, Hughes & Macdonald, 2013). These processes can affect plant composition and patterns (Boeken et al., 1995; Davidson & Lightfoot, 2008; Eldridge, Whitford & Duval, 2009).

Documented examples of ecosystem engineers, and their effects, are increasing, for example, porcupine diggings in the Negev desert redistribute resources (Boeken et al., 1995); soil invertebrates alter biogeochemical structure of soil (Jouquet et al., 2006); pocket gophers alter soil characteristics and vegetation patterns in North America (Reichman & Seabloom, 2002). However, cases of reintroducing lost ecosystem engineers and their consequent effects are rare. Examples include the return of beavers (*Castor canadensis* and *C. fiber*) which create meadows in otherwise forested landscapes, increasing species diversity (Rosell et al., 2005; Wright, Jones & Flecker, 2002) and wetland connectivity (Hood & Larson, 2015); reintroduced giant tortoises (*Geochelone nigra hoodensis*) which alter plant community dynamics and can assist recovery of an endangered cactus (Gibbs et al., 2014; Gibbs, Marquez & Sterling, 2008); the reintroduction of wild boar (*Sus scrofa*) into Scottish moorlands to reduce plant competition and aid re-establishment of woodland trees (Sandom, Hughes & Macdonald, 2013); and the reintroduction of digging marsupials which alter soil and resource heterogeneity (James & Eldridge, 2007).

Australia hosts a large number of native animals that dig extensively whilst either foraging or burrowing. After 150 years of habitat destruction and predation by introduced animals, the majority of these animals are now locally very rare or extinct (Fleming et al., 2014; Short, 1998). For most of mainland Australia, the loss of the digging animals may also mean the loss or reduction of the ecological processes associated with digging (Fleming et al., 2014). With no baseline data on the composition of healthy soil processes in south-eastern Australia pre-European settlement, the magnitude of the changes caused by a cessation of key ecological processes is unknown, but possibly substantial. However, it cannot be assumed that returning putative ‘ecosystem engineers’ to a degraded landscape will necessarily result in the desired improvement (Byers et al., 2006). Reintroduced species can also have unintended effects on other processes, for example, reintroduced omnivorous mammals in Australia have been shown to reduce the ecosystem engineering effects of termites (Coggan, Hayward & Gibb, 2016).

Quantification of digging (or biopedturbation) processes (such as the magnitude of disturbance) is not well understood in Australia’s mesic environments. The consequent effects of reintroduced engineers, are similarly not well understood, but are required to further understand how to adequately restore degraded landscapes. This study addressed a key knowledge gap as to the magnitude of digging per unit time of a native marsupial, and identified preferred digging locations in a degraded landscape. Our observations were of the reintroduced eastern bettong (*Bettongia gaimardi*) in temperate woodland that was historically the habitat for this species (Short, 1998), at the Mulligans Flat Woodland Sanctuary. The eastern bettong is a small member of the kangaroo family, which digs numerous small foraging pits (Johnson, 1994a). They previously occurred throughout south-eastern Australia, but have been extinct on the mainland since around 1906 (Short, 1998). They survive in the wild only on the island state of Tasmania. The eastern bettong is mycophagous, digging for and consuming the underground fruiting bodies of mycorrhizal fungi (Johnson, 1994b). Fungi makes up the bulk of the diet of the eastern bettong, but they also consume seeds, roots, leaves and some invertebrates (Taylor, 1992).

Assessment of the role of the bettong as a factor in the restoration of a degraded post-agricultural landscape would require:

1. Quantification of the nature and extent of the digging in relation to that of other digging species that have persisted or been introduced to the landscape.
2. Identification of habitat features (soil nutrients, microhabitat and vegetation structure) that are associated with animal diggings.
3. Determination of whether reintroduced bettongs dig in soil scalds and, therefore, facilitate recovery processes. NB—‘scalds’ are bare, eroded areas of texture-contrast soils which have lost A-Horizon (top-soil) material, are hard when dry and have little or no water infiltration (Tongway & Hindley, 2004). Scalds are an obvious and highly persistent manifestation of past poor grazing practices.
4. Measurement of the actual effects of the digging—whether erosion is mediated, native plant diversity maintained or increased, and the role of digging in promoting or suppressing exotic plants.

Our study addressed points 1–3 by recording the number, size and location of foraging pits over a 5-month period. This study was cross-sectional rather than longitudinal, and since bettongs breed and feed on fungi year-round, we presumed our observations accurately reflected bettong digging behaviour during this time. We observed foraging pits of the dominant ground-feeding vertebrates present. For each species, we assessed (1) the total number and volume of pits per ha, (2) broader landscape influences on pit density such as density of large grazers, vegetation structure and soil attributes, and (3) preferences for digging in different micro-habitats. The answers to these questions will address whether the eastern bettong could be an ecosystem engineer in this environment. Future investigations include the physical, chemical and biological changes resulting from digging activity (Jones et al., 2010; Jones, Lawton & Shachak, 1994).

## Methods

### Study site

The study was conducted in the 485 ha Mulligans Flat Woodland Sanctuary (henceforth ‘the Sanctuary’), a natural ‘outdoor laboratory’, near Canberra, Australia. It is the site of the long-term Mulligans Flat—Goorooyarroo Woodland Experiment to restore the critically endangered box-gum grassy woodland (Manning et al., 2011; Shorthouse et al., 2012) (http://www.mfgowoodlandexperiment.org.au; Australian National University Animal Experimentation Ethics Committee protocol A2011/017, and ACT Government project license LT2010417).

Mulligans Flat Woodland Sanctuary was fenced in 2009 to exclude predators and allow the re-introduction of locally extinct mammals such as the eastern bettong, which was reintroduced in 2012 (Batson et al., 2016; Shorthouse et al., 2012). The Sanctuary protects a regionally significant area of critically endangered box-gum grassy woodland in ‘good’ condition (ACT Government, 2004). However, it has suffered the same degrading processes as most Australian woodlands, in particular, a substantial loss of coarse woody debris, overgrazing by European rabbits (*Oryctolagus cuniculus*), domestic livestock and more recently by native kangaroos (*Macropus giganteus*) and altered fire regimes (Manning et al., 2011; Shorthouse et al., 2012). The effects of past management have resulted in soil that is eroded and compacted in parts of the landscape, and vegetation that has a high component of exotic species (McIntyre et al., 2010). Like other box-gum grassy woodlands, the area would previously have supported two or three species of digging marsupial mammals which are now locally extinct (e.g. the southern brown bandicoot (*Isoodon obesulus*) and the long-nosed bandicoot (*Perameles nasuta*), as well as the eastern bettong). The remaining native digging vertebrate animals include the echidna (*Tachyglossus aculeatus*) which digs for ants and termites, wallabies (small kangaroos; *Wallabia bicolor* and *M. rufogriseus*) which dig occasionally for fungi, and many native birds which dig for insects and underground storage organs of plants. The introduced European rabbit arrived in the study area in the late 1880s, and has been an abundant and frequent digger. The Sanctuary has been incorporated into a broader Mulligans Flat—Goorooyarroo Woodland Experiment, which focusses on restoration of a degraded woodland (Manning et al., 2011; Shorthouse et al., 2012). The greater project has focussed on the role of course woody debris (log) additions, grazing management of kangaroos and fire in ecological restoration, as well as aiming to restore influential or keystone species to restore ecological function, starting at the level of soil processes.

The climate is temperate with mean minimum temperature in winter of −0.1 °C and mean maximum temperature in summer of 28.0 °C. Mean annual rainfall of 616 mm is distributed evenly throughout the year (Bureau of Meteorology, 2015). The vegetation of the Sanctuary is predominantly grassy eucalypt woodland, with some sclerophyll eucalypt forest and grassland (Lepschi, 1993).

### Site-scale experimental design

The study was conducted in a subset of one ha sites (*n* = 36) within a larger experiment (*n* = 48 in Mulligans Flat). Detailed descriptions of the landscape and all treatments are given in McIntyre et al. (2010) and Manning et al. (2011).

Briefly, within the Sanctuary are 12 areas (we call polygons) representing the broad vegetation structure. This reflects the variation in density of trees and shrubs across the landscape (four categories: (i) high-tree high-shrub, (ii) high-tree low-shrub, (iii) low-tree high-shrub, (iv) low-tree low-shrub) calculated by whether the density at the site was above or below the mean for the entire Sanctuary (Treatment 1).

Half the polygons have reduced kangaroo density using fencing to contrast with the background numbers of eastern grey kangaroos (*M. giganteus*) which are very high in the district (Department of Territory and Municipal Servives, 2010). There were two kangaroo densities—high and low, where ‘high’ is similar to that outside the Sanctuary fence, and ‘low’ is approximately half this density, and approaching a more natural kangaroo density (Treatment 2).

Within each polygon are four one-hectare sites (200 × 50 m), each with randomly assigned Treatments 3 and 4. The four sites were randomly assigned a log addition treatment (Treatment 3). The addition of coarse woody debris was sourced from felled and fallen trees outside the experimental site and were placed (i) singly, (ii) in clumps, (iii) both clumped and singly or (iv) no logs added (Manning, Cunningham & Lindenmayer, 2013). Natural log volumes in each site were measured (A.D. Manning, 2018, unpublished data).

One of the four sites within each polygon was randomly selected to be fenced to exclude bettongs (control sites for other experiments) (bettong-exclusion treatment; Treatment 4). The re-introduction of eastern bettongs took place in 2012, 2 years prior to the observations reported here. The eastern bettong was re-introduced to test the hypothesis that it could enhance ecological processes through its digging of soils to find *mycorrhizal* fungi and other underground resources (Batson et al., 2016; Shorthouse et al., 2012).

Observations were made at the 36 sites in which the reintroduced bettongs had free access (Treatment 4). We used two different field methods (described below) to explore the three key questions:

1. large plots to determine pit densities and volume,
2. large plots and site-scale variables to determine the broader landscape influences on pit density, and
3. small plots to determine micro-scale preferences.

#### Sampling design to determine pit densities

Each one ha site (200 × 50 m) was permanently marked for other long-term research (Manning et al., 2011). At each site, four large plots (10 × 10 m) were established. Plots were placed 10–20 m from the midline, 60 m (left side) and 80 m (right side) from each end of the site. All fresh digging pits were counted in each large plot (see below). Therefore, we surveyed 144 plots (4 × 36) but aggregated data to site level (*N* = 36) to give a count per hectare.

We calculated densities and volumes of pits for each animal species that dug pits, and, where possible, calculated a digging rate per species using estimates of species population density. The population of bettongs within the Sanctuary was estimated to be 137 at the start of the study (July 2014), and increased to 186 at the end of the study period (November 2014) (mean 163 or 0.29–0.4 bettongs per ha) (A.D. Manning, 2018, unpublished data). Population estimates were achieved by four intense trapping sessions in February, August, and October 2014 and February 2015 and estimated using mark-recapture techniques.

Rabbit numbers were counted along a 13 km spotlight transect four times during the 5-month study. Rabbit numbers fluctuated between 1.4 and 2.8 rabbits per kilometre of spotlighting transect. The visibility zone on either side of the transect was visually estimated to be an average of 30 m, which gives estimates of 110–219 rabbits within the Sanctuary or 0.23–0.47 rabbits per ha. We acknowledge that rabbit estimates by spotlight transects can have poor accuracy (Fletcher, Moller & Clapperton, 1999; Twigg et al., 1998), and that our numbers are likely to be an underestimate. Rabbits in Mulligans Flat were subjected to intense control operations, and shooting estimates suggested the number of rabbits exceeded the number of bettongs at the time of the study. Here, we tentatively suggest a comparable number to bettongs (average 163) for ease of comparison.

We cannot provide accurate assessments of the populations of echidnas or birds. Echidnas were low in number (perhaps 30–50 individuals). Birds (predominantly white winged choughs, *Corcorax melanorhamphos*) were numerous.

#### Sampling design to determine site-scale preferences

We analysed pre-existing and new site data as possible explanatory variables for explaining pit densities in the large (10 × 10 m) plots. Data included:

- Soil variables measured in 2008: available phosphorus (mg/kg), total carbon (%), nitrate (mg/kg), total nitrogen (%) (McIntyre et al., 2010),
- Stem density and basal area of the dominant trees (*Eucalyptus* spp.) and shrubs (*Acacia* spp.) measured 2008 (A.D. Manning, 2018, unpublished data),
- Ground layer floristic composition; six groups derived from vegetation classification (McIntyre et al., 2010),
- Volume of naturally occurring logs measured in 2008 (A.D. Manning, 2018, unpublished data),
- Soil bulk density: a single soil core sample taken from each plot at the time of pit surveys, dried and weighed to determine bulk density.

#### Sampling design to determine microhabitat preference

In addition to the large plots at each site, we selected several small plots (a circle of one m radius) at particular habitat features. We selected (at random) one end of each site and from a permanent marker post 20 m into the site, identified the nearest examples of each of four microhabitat features (if available): (i) logs (added clumped and single logs, and naturally occurring logs, with and without the large tussock grass, *Rytidosperma pallidum*), (ii) tree (with *Rytidosperma*, without *Rytidosperma* or leaf litter, and with reasonable amounts of naturally occurring leaf litter), (iii) open area (with and without *Rytidosperma*) and (iv) scald (an area of at least one m diameter where the A-horizon soil was absent, and there was almost no vegetation beyond a cryptogamic crust). Within our study area, *R. pallidum* is indicative of drier areas with poorer soils (McIntyre et al., 2010) and is used in this study to determine whether eastern bettongs avoid lower productivity parts of the landscape.

The maximum number of microhabitat plots at a site was 12, but was as low as three at some sites because not all microhabitats were available. There was a total of 259 small plots. Each plot was a circle of one m radius, and all fresh pits were counted inside these plots (see below).

### Pit surveys

We visited all plots (both large and small) twice to determine the number and rate at which fresh pits were dug in the period between the two surveys. On the first visit, we identified all existing pits within the plot, and marked these by placing a small purple pebble in the bottom of the pit. On the second visit 2 weeks later, we counted all new pits (those without pebbles) and measured their dimensions to estimate the volume of material removed. Using this technique, we could ensure that all pits were fresh (up to 2 weeks old), and, therefore, we could calculate the rate of digging per unit time. For most pits we were able to identify the animal responsible with medium to high confidence (Supplementary Information), leaving only 13% and 15.6% of all pits for which we were uncertain of the identity of the digger (microhabitat level and site level assessments, respectively). These unknown pits and those dug by kangaroos and wallabies were classed as ‘other’.

Field work was conducted from July (mid-winter) to November (late spring), 2014. Due to the long duration of fieldwork, we included an explanatory variable ‘season’ (three categories: late winter (July–early September), early spring (September–October) and late spring (October–November) to determine if the digging pattern differed over this time frame.

### Data handling and statistical analyses

All statistical computation was undertaken using GenStat (version 17).

#### Site level

We calculated the density of fresh pits dug by bettongs, rabbits and birds, by aggregating the number of pits in each of the four large plots to derive an estimate of pits per hectare. We tested the influence of the experimental treatments (Treatments 1–3 above) and the additional environmental variables ((a)–(e) above) on pit density of bettongs, rabbits and birds. There were insufficient data for echidnas for further analyses.

We fitted a negative binomial regression model (due to over-dispersion and the clumped nature of the data) on the number of pits per hectare as a function of broad vegetation structure (high and low tree and shrub density), kangaroo exclusion and log treatment. Covariates included: stem counts of *Acacia* spp. and *Eucalyptus* spp. (count per ha), the basal area of *Acacia* and *Eucalyptus* (m^2^ per ha), understorey group, natural log volume, soil available phosphorus (Colwell), total carbon, total nitrogen, nitrate and bulk density. We used backwards stepwise processes for assessing the importance of variables. We present several alternative models rather than selecting a single best model.

#### Microhabitat level

Only bettong pits were analysed for the relationship with microhabitat features due to insufficient data for the other species. We examined linear logistic regression models using binary data (i.e. presence or absence of at least one fresh pit at a plot). Although our focus was on the effect of microhabitat type, due to the nested design of our experiment we included vegetation structure class (at the polygon scale) and natural log volume (at the site scale) as possible explanatory variables. We also included a variable, ‘season’, to account for the duration of field work over the seasons (late winter to late spring) and the growing population of bettongs during this time. We used a backwards stepwise process for assessing the importance of variables. Predictions from the regression model were calculated for each explanatory variable by averaging over the levels of the other factors in the model.

We also considered grassland type and tree species as alternative explanatory variables, by conducting a separate linear logistic regression model on binary data where the explanatory variable was grassland type (*R. pallidum* present or absent) (note this excluded trees with litter and plots with scalds), and another separate model of tree plots only, where we considered the tree species (*E. melliodora,* vs. *E. blakelyi* vs*. E. macrorhyncha*, vs. other).

## Results

Bettongs dug small pits of average 4.9 by 3.9 cm across, with 3.1 cm depth (Supplementary Information) giving an average volume of 36 cm^3^. The pits of rabbits were shallower but wider (10.0 × 6.1 wide, 2.2 cm deep, volume 89 cm^3^). Birds, predominantly white-winged choughs, dug small narrow but highly variable pits (3.1 × 2.2 wide × 2.3 deep, volume 15.6 cm^3^) while echidnas dug very large pits (11.4 × 8.1 wide × 3.8 deep, volume 224 cm^3^).

### Magnitude of digging

**Figure 1:**
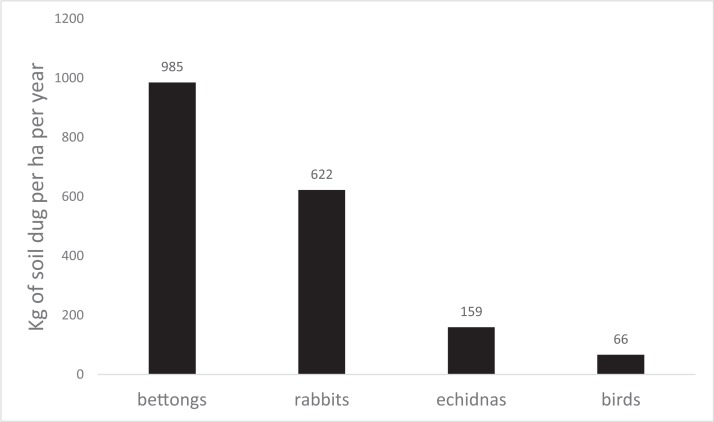
Soil movement by different species. Soil moved by four different kinds of animals in kg per ha per year, at the population present in the Sanctuary at the time. These estimates are based on surveys of 144 (10 × 10 m) plots in the Mulligans Flat Sanctuary over the period July–November of 2014.

The population of 137–186 bettongs dug on average 75.4 pits per ha per night, summing to 985 cubic m per year. Bettongs dug approximately 218 pits/night/individual, or 7.8 kg/night/individual. Bettong digging accounted for more soil turnover than rabbits, echidnas and birds combined (Fig. 1). Nevertheless, the soil turnover by rabbits was more than half that of bettongs. The pit density per individual bettong appears to be much greater than that for rabbits (Table 1), if our assumption of similar population size is correct. The impact of bird digging was limited by the small size of their pits, whereas echidna pits were very large and their soil volume turnover was disproportionately large compared to the number of pits.

**Table 1:** Digs per species.

	Bettongs	Rabbits	Birds	Echidnas
Proportion of total pits (%)	55	15.6	11.3	1.8
Pits/ha/night	75.4	19.4	9.5	1.8
Average pit surface area (cm^2^)	16.1	57.0	16.9	105
Average pit volume (cm^3^)	35.9	89.3	15.6	224
Average pit mass (g)	35.8	87.9	16.4	236
Surface area/ha/night (cm^2^)	1,214	1,106	160	189
Volume/ha/night (cm^3^)	2,707	1,732	148	403
Mass/ha/night (g)	2,699	1,705	156	425
Pits/night/individual	218	56.3		
Volume per individual animal (m^3^/individual/year)	2.85	1.8		
Mass per individual animal (kg/individual/year)	2,848	1,806		

**Note:**

Number of fresh pits (<2 weeks old) per species (assessed at site level), presented as density, volume and mass. Values per hectare per night are for the total populations found in the reserve at the time (average of 0.34 animals per hectare for bettongs and rabbits, unknown population for birds and echidnas).

Preliminary information from a related study on pit duration, from 170 artificial pits of bettongs and rabbits (hand dug pits matching the average dimensions taken from 1,518 bettong and 432 rabbit pits), indicate that there is no difference in longevity between the pits of the two species, and that after 2 years, only 22% of pits are completely full (C.E. Ross, 2018, unpublished data).

### Site level influences on pit density

**Figure 2:**
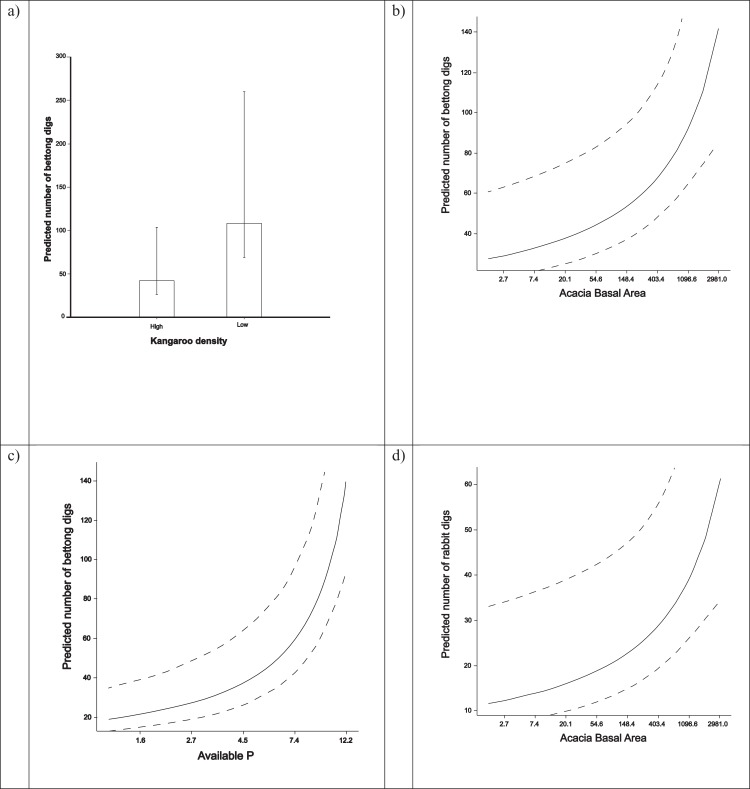
Predicted density of fresh pits relative to significant drivers at the site scale, all showing the mean and associated 95% confidence intervals, derived from models of field data. All models used a backwards stepwise regression model of the number of pits per hecture. (A) The number of bettongs pits per hectare at two kangaroo densities (*P* = 0.034), (B) the number of bettong pits per hectare by the basal area of Acacia (native small tree) (*P* = 0.005), (C) the number of bettong pits per hectare by available soil phosphorus (P) (*P* < 0.001) and (D) the number of rabbit pits per hectare by the basal area of Acacia (*P* = 0.017).

Analysis of the density of fresh pits in the site-level survey showed significant associations with a number of variables, presented as separate competing non-nested models. Bettongs pits were more common where kangaroo grazing was reduced (*P* = 0.03), where *Acacia* basal area was greater (*P* = 0.005) and where available phosphorus in the soil was higher 5 years previously (*P* < 0.001) (Table 2; Fig. 2). When combined, our model included *Acacia* basal area (log transformed) and available phosphorus (log transformed). In contrast, there were no significant effects of log treatment, volume of natural logs, Eucalypt stem density, season, ground layer floristic composition, soil nitrogen, soil carbon or bulk density on the number of bettongs pits/ha.

**Table 2:** Model results of digs.

Species	Variables	d.f.	Deviance	*P*
Bettong	Log(available *P*)	1	17.1	<0.001
	Log(basal area Acacia)	1	12.5	0.005
	Kangaroo exclusion	1	7.6	0.034
Rabbit	Log(basal area Acacia)	1	7.6	0.017
	Kangaroo exclusion	1	4.7	0.066
Birds	Log(available P)	1	7.5	0.006
	Log treatment	3	9.7	0.021

**Note:**

Summary of analysis of deviance for variables that had a significant effect on the number of fresh pits per hectare assessed at the site level. Note that each of these variables were modelled independently, with each model presenting a single significant variable. Probabilities for the bettong and rabbit models are approximate *F*, and for the bird model approximate Chi.

Fresh rabbit pits were more common where *Acacia* basal area was greater (*P* = 0.017, Table 2; Fig. 2), and there was a near significant increase in rabbit pits where kangaroo grazing was reduced (*P* = 0.07). These two different effects were detected only when effects were modelled separately. The number of fresh rabbit pits per ha was also not influenced by log treatment, natural log volume, Eucalypt stem density, season, ground layer floristic composition, or any soil chemicals or characteristics.

Analysis of fresh bird pits indicated they were fewer where available soil phosphorus was higher 5 years previously (*P* = 0.006). When modelled alone, there was an effect of logs on the number of bird pits (*P* = 0.02), whereby they were more frequent in sites with dispersed logs than in the other log treatments but when available phosphorus was included in the same model, the log treatment was no longer significant. No other variables were significant predictors in our models for bird pits.

### Preferences for digging in different micro-habitats

**Figure 3:**
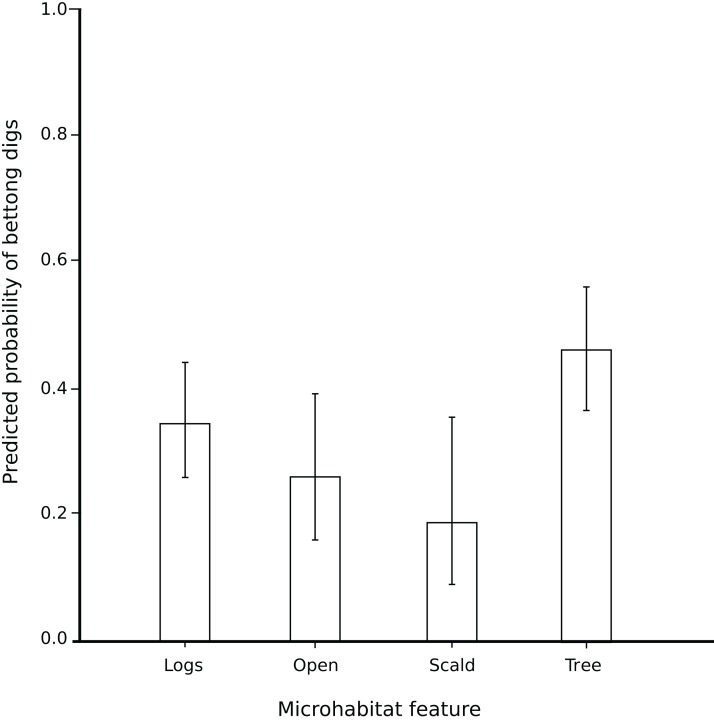
Bettong digs in microhabitats. The mean probability (and 95% confidence intervals) of a bettong dig occurring across four kinds of microhabitat feature. The probability of a dig occurring differed by microhabitat type (*P* = 0.008). ‘Scalds’ had a significantly lower chance of a dig occurring than the remaining three microhabitat types, and when ‘scalds’ were removed from the model, ‘trees’ had a significantly higher chance of a dig than the ‘logs’ and ‘open’.

Microhabitat type did influence the probability of pit occurrence (*P* = 0.008), even after accounting for the fixed effects at the higher level (vegetation structure class and season). There were no significant interactions between these terms (Table 3; Fig. 3), indicating that bettong digging at different microhabitats did not change in the different seasons and was not influenced by overstorey vegetation density.

**Table 3:** Model results on digs microhabitats.

Variables	d.f.	Deviance	*P*
Season (adjusted for vegetation structure class and microhabitat feature)	2	13.1	0.001
Vegetation structure class (adjusted for season and microhabitat feature)	3	19.9	<0.001
Microhabitat feature (adjusted for season and vegetation structure class)	3	11.9	0.008

**Note:**

Summary of analysis of deviance for variables that had a significant effect on the probability of a fresh pit occurring in plots centred on microhabitat features (log, tree open, scald). Probabilities are approximate Chi.

We were particularly interested in how scald plots compared with other plot types so we conducted a further pair-wise analysis which showed that scald plots had a significantly lower probability of pits occurring than the other three microhabitat features (i.e. the mean of ‘logs’, ‘open’ and ‘trees’) (*P* = 0.02). We then removed ‘scald’ from the analysis, and the predicted probability of a bettong pit at a ‘tree’ was significantly greater than the other two microhabitat features (‘logs’ and ‘open’) (*P* = 0.02). Although bettong digging in the scald plots was the least frequent of the four categories, the fact that pits were present at all is notable. Half the pits in scald plots were near but not on the actual scalded areas and half were directly into scalded soil surface. Bettongs were the only species observed to dig in scalds during our study.

Vegetation structure class had the strongest effect on pit occurrence (*P* < 0.001) with ‘high-tree, low-shrub’ sites showing the greatest probability of a pit occurring (predicted probability of a pit of 0.52) and ‘low-tree, low-shrub’ sites having the lowest probability of a pit occurring (predicted probability of a pit of 0.21). Season significantly affected the probability of a pit occurring (*P* = 0.001) with fewer occurrences during the early spring season than either before or after this time (Fig. S1).

The probability of fresh bettong pits was not influenced by whether the grass layer was dominated by *R. pallidum* or not (*P* = 0.809) or by the identity of the tree above the tree plots (*P* = 0.540).

## Discussion

### The magnitude of digging

Our data have confirmed that the recently re-introduced eastern bettong has now become the most significant cause of soil perturbation in our woodland Sanctuary, relative to the other major diggers, the introduced European rabbit, the echidna and ground-foraging birds. At the population densities present in the Sanctuary, bettongs can be predicted to turn over 985 kg of soil per ha per year, which was much greater than that for rabbits (622 kg/ha/year), echidnas (159 kg/ha/year) or birds (66 kg/ha/year). This results from the large number of pits dug by bettongs, which were smaller in size than those of rabbits and echidnas, but larger than those created by birds. We acknowledge that extrapolating from a 5-month study to yearly estimates assumes consistent digging behaviour throughout the year. However, yearly estimates are a common comparison due to differences in time frames for different studies, and our study duration is much longer than most.

The volume of soil moved is a function of the density of the animals at a particular time as well as the nature of the excavations. In the case of echidnas, density of the animal was not controlled by humans or other known predators, and the density probably approximated the carrying capacity for this species. Rabbit densities in the region fluctuate wildly depending on rainfall, disease and predation pressure. The rabbit population in the Sanctuary was subject to an intensive control program, but terrestrial predators were absent. The rabbit density was within the range of that outside the Sanctuary. At the time of writing (June 2018), rabbits have been eradicated from the Sanctuary to protect the vegetation, so the bettong is now the major bioturbation agent.

It is difficult to meaningfully compare the activities measured in this study with that of other ecosystems, since population density of animals can differ substantially for many reasons and benchmarks for appropriate densities mostly do not exist. Most other studies were designed for within-site comparisons, which does not require information on population density, thus many studies did not attempt a population estimation (Eldridge & Kwok, 2008; Johnson, 1994a; Lamont, Ralph & Christensen, 1985). Additionally, digging rates reported in many published studies did not calculate a time period over which the pits were created (Claridge, Cunningham & Tanton, 1993; De Villiers & Van Aarde, 1994; James & Eldridge, 2007; Johnson, 1994a; Mallick, Driessen & Hocking, 1997). This is important because longevity is likely to be different in different vegetation communities and with different rainfall and aspect (N. Munro, 2012 to 2014 Mulligans Flat, 2000 to 2003 Arid Recovery, personal observation). Thus, only digging rates per unit time can be compared, and studies need to be designed specifically to calculate this, such as we have done. However, the summary of comparable studies (Table 4) suggests that the eastern bettong are very active diggers, digging at the highest rate recorded (pits per individual), however, the volume of soil turnover per individual is similar to the woylie (*Bettongia penicillata*, a related species in forests of Western Australia), but much less than digging mammals in the Australian arid zone (bilby and burrowing bettong) which live in sandy substrates. The arid zone species dug fewer (120 pits/individual/night), but much larger pits (approximately 10 times as large as that of the eastern bettong) (Newell, 2008).

**Table 4:** Digging rates global comparisons.

Species	Pits per ha per year	Pits per individual per night	Soil turnover, tonnes per individual per year	Ecotype	Country	Reference
Eastern bettong, *Bettongia gaimardi*	27,521	217.6	2.8	Temperate woodland	Australia	This study
European rabbit, *Oryctolagus cuniculus*	7,081	56.3	1.8	Temperate woodland	Australia	This study
Greater Bilby, *Macrotis lagotis* plus Burrowing Bettong, *Bettongia lesueur*		120	30	Arid shrubland	Australia	Newell (2008)
Woylie (*Bettongia penicillata*)	5,000–16,000	38–114	2.7–9.7	Temperate woodland	Australia	Garkaklis, Bradley & Wooller (2004)
Southern brown bandicoot		45	3.9	Temperate woodland	Australia	Valentine et al. (2012)
Heteromyid rodents	111,600			Arid shrubland	USA	Steinberger & Whitford (1983)
Indian crested porcupine (*Hystrix indica*)	300–1,800			Desert	Israel	Alkon (1999)
Aardvark (*Orycteropus afer*)	239			Semi-arid shrubland	South Africa	Dean & Milton (1991)
Bat-eared fox (*Otocyon megalotis*) and Cape fox (*Vulpes chama*)	109.8			Semi-arid shrubland	South Africa	Dean & Milton (1991)

**Note:**

A comparison of digging rate of eastern bettongs and rabbits in our study to that of other similar species presented as the digging rate (for unknown population), digging rate per individual, and soil turnover in mass. Presented are only those studies with a digging rate per unit time.

### Could eastern bettongs enhance soil processes?

Our study environment contains many highly degraded areas that have been compacted and eroded by livestock and cultivation, which are represented in this study as scalds (McIntyre et al., 2010, 2014)—areas with few plants, low water infiltration and a smooth surface resisting the lodgement of litter and seeds (Tongway & Ludwig, 2010). Similarly degraded areas are also found between the tussocks in some grassed areas. In many systems, scalds tend to persist even if the causative agents are removed (Tongway & Ludwig, 2010). McIntyre et al. (2014) noted no change in amount of bare ground in our study site between 2007 and 2011, despite reduced native grazing pressure and livestock removal. Recovery is likely to be incremental and extremely slow. The soil perturbation activities of eastern bettongs may have the potential to hasten the recovery of these areas, since bettongs were observed to dig in scalds (albeit at a lower rate than in other areas). No other animal was observed to dig at scalds in our study, so even the low rate of disturbance of scalds may have a significant effect over the long term. We do not know what food resource bettongs were seeking or acquiring from their digging in and around scalds. By establishing that eastern bettongs dug not only in areas of good soil functioning (e.g. fertile soils, under trees, near logs) but also in open and eroded areas, then it is possible they could play a role in the enhancement of soil processes in areas previously degraded by livestock and cropping. Additionally, the presence of digging in areas dominated by *R. pallidum* (associated with the least fertile sites McIntyre et al., 2010) suggests that eastern bettongs were not avoiding areas of low productivity, thus increasing the probability that they could play an important role in soil restoration in these areas.

At a population density of 0.23–0.37 individuals per hectare, eastern bettongs were excavating in the order of 985 kg of soil/ha/year, which has the potential to significantly affect the soil, nutrient and vegetation dynamics of the grassy woodland ecosystem. Pits, although small, persisted for a long time (we found that only 22% of pits were completely full after 2 years).

Most studies of digging soil engineers and their effects on soil processes are from arid and semi-arid regions. This study adds to the limited information for temperate regions of Australia (Garkaklis, Bradley & Wooller, 2004; Johnson, 1994a; Valentine et al., 2012). It is important to study the role of ecosystem engineers in a range of environments, from physically harsh to benign, because the underlying processes may be different (Crain & Bertness, 2006). In harsh environments, soil engineers can ameliorate the harsh environment by creating safe refuges, for example, burrowing bettongs create pits for seed germination (James, Eldridge & Moseby, 2010), whereas in benign environments, soil engineers may relieve biotic limitations such as predation and competition (Crain & Bertness, 2006). The role of soil engineering is yet to be fully tested in our environment.

In arid and semi-arid regions, pits have been shown to trap leaf litter (Eldridge & James, 2009; Eldridge & Mensinga, 2007; James & Eldridge, 2007; James, Eldridge & Hill, 2009; Newell, 2008) and to be sites of high water infiltration (Garkaklis, Bradley & Wooller, 1998; Laundre, 1993; Martin, 2003). These abiotic changes allow microbial activity to increase which accelerates the breakdown of leaf litter and incorporation of nutrients. Thus, pits become sites of concentrated nutrients (Eldridge & Mensinga, 2007; James, Eldridge & Hill, 2009) (further abiotic change). Both the capture of seed and the nutrient changes associated with the capture of leaf litter can create refuges for arthropods (Eldridge & Mensinga, 2007) or sites of increased germination of seedlings (Boeken et al., 1998; James, Eldridge & Hill, 2009; James, Eldridge & Moseby, 2010; Martin, 2003; Sparkes, 2001) (biotic change). This can create larger-scale changes to xeric vegetation systems (Alkon, 1999; Boeken et al., 1995; Davidson & Lightfoot, 2008; Gutterman, Golan & Garsani, 1990; Reichman & Seabloom, 2002). These processes are still to be demonstrated in our study area.

### Patterns of digging behaviour

We found a preference for digging near eucalypts, possibly for the fruiting bodies of mycorrhizae which make up a substantial portion of their diet (Taylor, 1992). However, bettongs also dug substantially in open areas which suggests they may facilitate the spread of mycorrhizae fungal spores from treed environments to areas where young eucalypts may regenerate (Lamont, Ralph & Christensen, 1985). Our results differed from that of Johnson (1994a) in that bettongs were not particularly more attracted to thickly treed areas as compared to more open woodland.

While bettongs did not respond to tree density, pit density was higher in the presence of shrubs, primarily *Acacia* species. Bettongs are known to consume the exudate of *Acacia* spp. (Taylor, 1992 and K. Grarock, 2015, personal communication) and Johnson (1994a) observed most eastern bettong pits around both *Eucalyptus* and *Acacia* trees. In our woodlands, *Acacia* thickets may provide additional attractions of insect larvae or protection from predators such as raptors and owls.

We found that bettongs did not preferentially dig near logs, which was somewhat unexpected given the microclimate is potentially favourable for foraging. Soil moisture and plant biomass are greater near logs (Goldin & Brookhouse, 2015), and beetle abundance and species richness is greater at logs than at nearby open ground (Barton et al., 2009, 2011). We expected these might be positive factors in the provision of food.

Bettongs dug more pits where levels of available phosphorus in the soil were higher. Initially, this seems contrary to other studies which suggest eastern bettong habitat is typically on soils of low fertility (Johnson, 1994a; Rose, 1986), with Johnson (1994a) specifically suggesting soils of low phosphorus. Claridge & Barry (2000) also found that similar species, bandicoots and potoroos, dug more frequently on soils of lower fertility, in a forested ecosystem. However, the soils in our study area are low in phosphorus generally (typically five to eight mg/kg), and it appears bettongs are responding to small gradients within this narrow and low range. We do not know the mechanism behind this, but tentatively suggest this may reflect the slightly greater soil fertility found in treed areas (Prober, Thiele & Lunt, 2002). Interestingly, birds (predominantly white-winged choughs) also responded to available phosphorus, but with a negative relationship (more bird pits where phosphorus was lower). We do not know the mechanisms here either, but note curiously that bettongs appeared to dig where other abundant extant species did not.

### Can rabbits fulfil the engineering role of bettongs?

The managed replacement of rabbits with bettongs in the Sanctuary raises the question of the extent to which the rabbit has played a substitute role as a soil engineer during the century or more that the eastern bettong has been absent, and the rabbit present. Rabbits create extensive burrow systems which are considered to have a negative effect on soil properties in semi-arid vegetation (Eldridge & Koen, 2008; Eldridge & Simpson, 2002). In the Australian arid zone, rabbit foraging pits were fewer, and trapped substantially less leaf litter and seed than those of native diggers (James & Eldridge, 2007; James et al., 2011) and did not enhance soil microbes (Eldridge et al., 2016). Conversely, the pits of native diggers were larger than those of rabbits (James, Eldridge & Hill, 2009) which may have accounted for some of this difference. In our study, rabbits did not dig to the same extent as bettongs, but their pits were slightly larger, although shallower, than that of bettongs.

There are overarching reasons to remove rabbits from Australian woodlands due to their damage to native vegetation (Auld, 1995; Cooke, 2012; Hall, Specht & Eardley, 1964; Munro, Moseby & Read, 2009) and to replace them with native species such as eastern bettongs (including conservation of the eastern bettong itself). Rabbit populations are controlled over large areas of Australia although rabbit eradication is not feasible at present, other than in well-managed fenced sanctuaries. While rabbits may have provided less of a soil disturbance role compared to the original native species, their removal would necessitate a reciprocal reintroduction of native diggers to ensure the bioturbation process is maintained. It is not known at this stage if the possible beneficial foraging digging by rabbits outweighs the negative impact on vegetation.

### Further investigations relevant to the role of eastern bettongs in woodland dynamics

We have established that eastern bettongs can, and do, forage in degraded, scalded soils. However, the effectiveness of these pits in capturing water and plant materials, and in restoring soil functions such as rainfall infiltration and nutrient cycling, still requires research. The effects of digging by the eastern bettong may not be apparent for many years or even decades. Only with this further information can we decide if the eastern bettong is, indeed, an ecosystem engineer of significant importance (Jones et al., 2010).

Whilst the eastern bettong has been reintroduced to potentially enhance lost or reduced processes, we must also appreciate that the soils of our study area are different today than in pre-European times. Australian soils suffered substantial depletion in the first decades after agriculture began in the 1800s, but have since been stable (Gale & Haworth, 2005). This stable degraded state may make restoration more difficult or unpredictable (Lunt et al., 2007; Prober, Thiele & Lunt, 2002). Pre-European bettong populations may have been different to that found in our reserve due to the presence of historical predators, and bettongs may have had different digging requirements within previous non-degraded soils.

We observed bettong digging behaviour over a 5-month period only. We observed a lower occurrence of pits in early spring when modelled alone for microhabitat data only, but otherwise no statistically significant seasonal changes to digging behaviour were observed. At this time, bettongs were observed to dig the geophyte (*Wurmbea dioica*) consuming the underground corm. This may have been a more concentrated form of nutrition than that available in other seasons. Another explanation is that alternative above-ground food sources may have become available in early spring. We acknowledge there may be changes at other times of the year, or interannual variation due to shifts in diet. The eastern bettong is one of several species of digging marsupial that previously occurred in our study area. A combination of digging species may have additional or complementary effects on soil processes (see Davidson & Lightfoot, 2008). Thus, the historical level of bioturbation in our study area may have greatly exceeded that which we observed.

## Conclusion

Our study has established that eastern bettongs create distinctive excavations during foraging, moving almost 1,000 kg/ha/year for the total population of animals present, or 2.8 tonnes/individual/year of soil, far exceeding the other native diggers in the study area (birds and echidnas) and also exceeding the feral rabbit, which has geographically replaced the bettong over the past 100 years. Given that eastern bettongs occurred throughout the extent of box-gum grassy woodlands, this would have equated to a vast amount of soil movement per annum (along with associated ecosystem effects) before they became functionally extinct. The location of foraging pits of the eastern bettong suggests this species is active, not only under *Eucalyptus* canopies (where their main food, hypogeal fungi, occurs), but also in open grassy areas. We also recorded limited foraging activities in degraded, scalded areas, providing evidence that the eastern bettongs have the potential to enhance recovery of soil processes in areas damaged by past farming activities.

### Implications for practice

- Eastern bettongs dig frequently throughout their environment, causing substantially greater bioturbation than other extant native species, and more than the introduced European rabbit which geographically replaced bettongs 100 years ago. Removing feral rabbits and reintroducing eastern bettongs may return the naturally high bioturbation rates of Australian woodlands.
- Eastern bettongs were the only species observed to dig in highly degraded soil scalds. Thus, they may have the potential to facilitate the restoration of these areas, which are otherwise persistent.
- Quantifying the magnitude and persistence of pits dug by native wildlife is the first step towards determining if the eastern bettong plays a significant role as an ecosystem engineer.
- Bettongs could be reintroduced to sites dominated by Acacias, given the extra resources they provide for bettongs. In the temperate regions of Australia, dense Acacia shrublands are usually indicative of natural regeneration after substantial degradation and clearing, or fire. Such sites, while considered degraded, may be suitable for eastern bettongs.

## Supplemental Information

10.7717/peerj.6622/supp-1 Supplemental Information 1 The probability of a bettong dig occurring by season. The mean probability (with 95% confidence intervals) of a bettong dig occurring across three time periods: Jul–Aug (‘Winter’), Sept–Oct (‘Early spring’) and Nov–Dec (‘Late Spring’) for microhabitat data only. The probability of a dig occurring differed by season (P=0.001).

10.7717/peerj.6622/supp-2 Supplemental Information 2 Descriptions of digs within this study. Table 1. Dimensions and descriptions of the pits encountered in the study by different species.

10.7717/peerj.6622/supp-3 Supplemental Information 3 Dataset for bettong dig project. Data for bettong dig project presented as four worksheets on dig counts for large and small plots, soil volume and mass data, and data on the number of digs per species.

## Additional Information and Declarations

### Data Availability

The following information was supplied regarding data availability: The raw measurements are available in File S1 which provides four sets of data on counts in big plots and small plots, soil volume and mass information and counts per animal species.
