# Supplementary figures and images for: Returning a lost process by reintroducing a locally extinct digging marsupial
Source: PeerJ. 2019 May 27;7:e6622. doi: 10.7717/peerj.6622 (PMC6542348; doi:10.7717/peerj.6622)

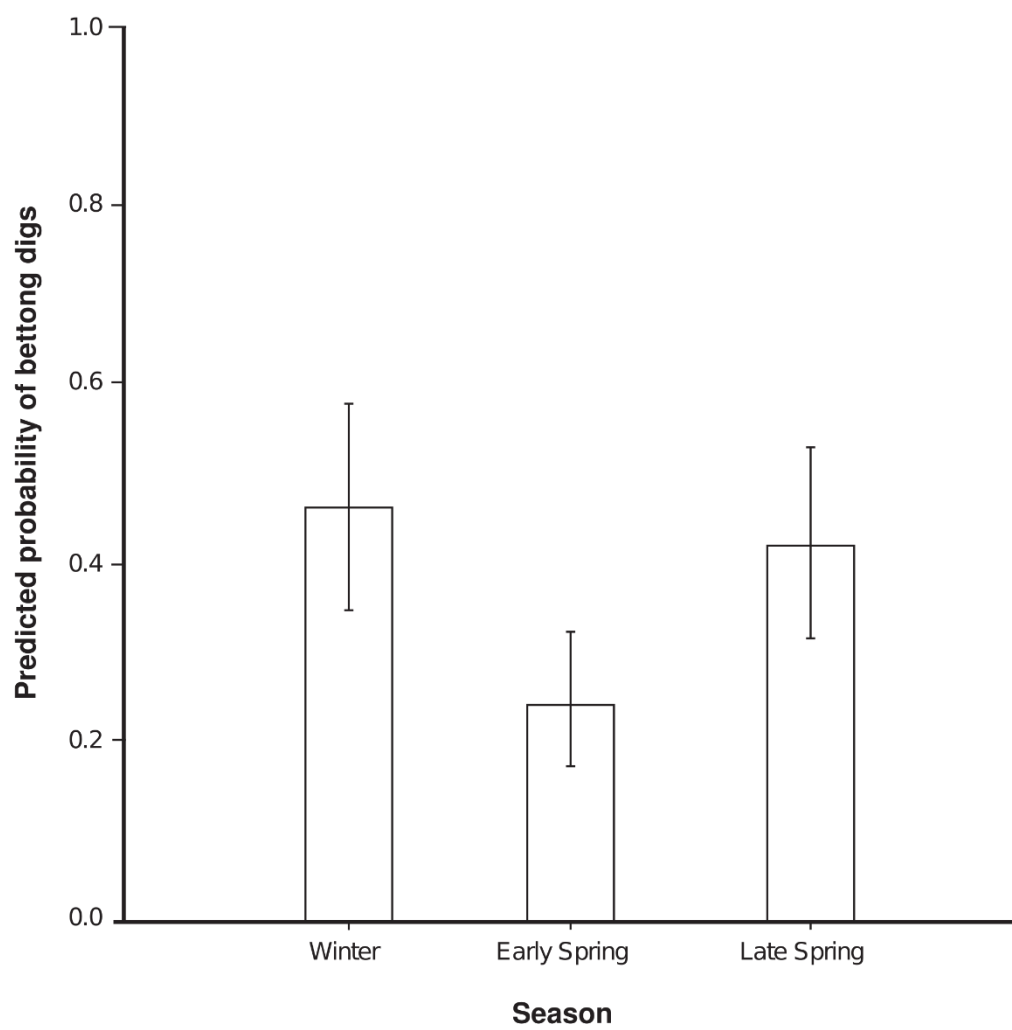

Supplement: Supplemental Information 1 — The mean probability (with 95% confidence intervals) of a bettong dig occurring across three time periods: Jul–Aug (‘Winter’), Sept–Oct (‘Early spring’) and Nov–Dec (‘Late Spring’) for microhabitat data only. The probability of a dig occurring differed by season (P=0.001). [file peerj-07-6622-s001.pdf]
