# Supplementary material for: Returning a lost process by reintroducing a locally extinct digging marsupial
Source: PeerJ. 2019 May 27;7:e6622. doi: 10.7717/peerj.6622 (PMC6542348; doi:10.7717/peerj.6622)
Supplement: Supplemental Information 2 — Table 1. Dimensions and descriptions of the pits encountered in the study by different species. [file peerj-07-6622-s002.docx]

Supplementary Information

Table 1. Dimensions and descriptions of the pits encountered in the study by different species.

| Species | Dig dimensions (length x width x depth) (cm) | Mean dig volume (cm^3^) | Description | Photo |
| --- | --- | --- | --- | --- |
| Bettong | 4.9 x 3.9 x 3.1 | 35.9 | Small discrete pit with spoil heap on one side, sometimes into the roots of grasses or shrubs | 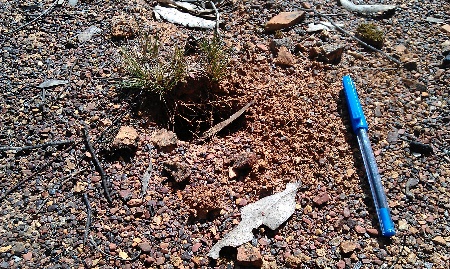 |
| Rabbit | 10.0 x 6.1 x 2.2 | 89.3 | Wide shallow, smooth depression, with spoil heap on one side, often with faeces present | 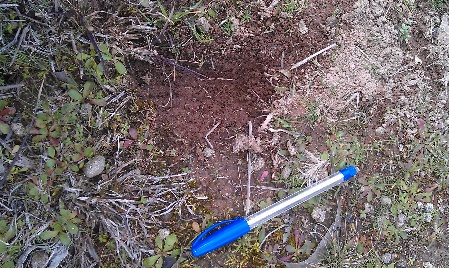 |
| Chough | 2.9 x 2.1 x 2.3 | 14.4 | Narrow deep pit with soil scattered in all directions. No spoil heap | 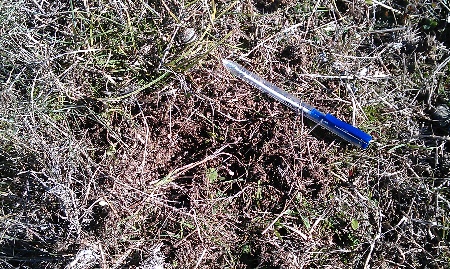 |
| Cockatoo | 6.0 x 2.0 x 2.5 | 31.6 | Long deep gouge with balls of soil cast aside | 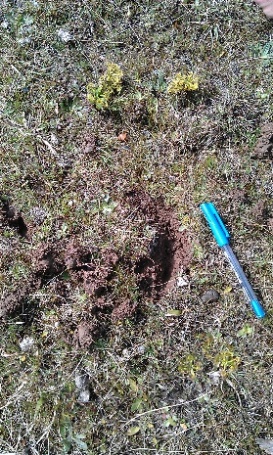 |
| Echidna | 11.4 x 8.1 x 3.8 | 224.1 | Large untidy pit with soil pushed forward into mounds, no neat spoil heap | 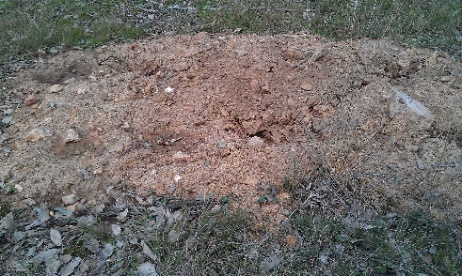 |
